# Comparative Genomic Analysis of *Acheta domesticus* Densovirus Isolates from Different Outbreaks in Europe, North America, and Japan

**DOI:** 10.1128/genomeA.00629-13

**Published:** 2013-08-15

**Authors:** Hanh T. Pham, Hajime Iwao, Jozsef Szelei, Yi Li, Kaiyu Liu, Max Bergoin, Peter Tijssen

**Affiliations:** INRS, Institut Armand-Frappier, Laval, QC, Canadaa; Marinpia Nihonkai 5932-445, Nishifunami, Niigata, Japanb; Bioengineering Department, Wuhan Bioengineering Institute, Yangluo, Wuhan, Hubei, People’s Republic of Chinac; College of Life Sciences, Central China Normal University, Wuhan, Hubei, People’s Republic of Chinad

## Abstract

The first densovirus from a cricket, *Acheta domesticus* densovirus (AdDNV) (*Parvoviridae*), was isolated in Europe in 1977 and has been studied previously. We compared seven additional AdDNV genomes isolated from 4 other European outbreaks, 2 major North American outbreaks, and a Japanese outbreak. Phylogenetic analysis suggested that the 2009 Japanese and North American outbreaks were not related.

## GENOME ANNOUNCEMENT

The United States cricket pet food industry supplies billions of live crickets (*Acheta domesticus* L.) per year to pet stores and individuals. This industry has been devastated by epizootic *Acheta domesticus* densovirus (AdDNV) outbreaks since 2009 (1). Alternative, presumably virus-resistant, field cricket species have been introduced and led to widespread United States (and European) distribution of exotic *Gryllus* species, such as the naturally widespread African, European, and Asian “black cricket,” *G. bimaculatus*, and the previously unknown red cricket, *G. locorojo*, despite existing United States federal regulations to prevent such movement (2).

Previously, AdDNV outbreaks have occurred in Europe, and a small, contained one occurred in an operation in the Southeastern United States (3). The isolate from this U.S. outbreak, preserved in paraffin, was sequenced and was found to be identical to the European isolate from 1977 (1). The 1977 isolate (4) was cloned and sequenced and its expression strategy studied (5). In particular, expression of the structural proteins was found to differ from those of other parvoviruses, since two large open reading frames (ORFs) were spliced and VP2 was not completely contained within VP1, i.e., structural proteins did not form a nested set of N-extended proteins like those of other densoviruses. The *Blattella germanica* densovirus (BgDNV) was found to have adapted a similar strategy (6).

AdDNV samples were obtained from different outbreaks: June 2004 (AdEu04), July 2006 (AdEu06), May 2007 (AdEu07), and August 2009 (AdEu09), all in Germany; September 2009 (AdNA09) in the United States and Canada; and December 2012/January 2013 in Japan (AdJP12). Moreover, AdDNV was isolated from different cricket species, *Gryllus sigillatus* in September 2012 in Canada (GsCa12) and *Gryllus locorojo* in the United States in March 2012 (GlUS12). For all isolates, DNA samples were isolated as previously described (5). Primers (5) were designed on the sequence obtained previously for the 1977 AdDNV isolate from Europe (GenBank accession number HQ827781), so that overlapping amplicons could be obtained, whereas termini were cloned as described previously (7, 8).

At least two complete clones of each isolate were sequenced in both directions, and four times at locations of discrepancies, using Sanger’s method and the primer-walking method as described previously (9). Contigs were assembled by use of the CAP3 program (http://pbil.univ-lyon1.fr/cap3.php/) (10). Like some other densoviruses (11–16), AdDNV was found to have a broad host range, infecting at least *Acheta domesticus*, *Gryllus locorojo*, and *Gryllus sigillatus*. In fact, AdNA09 and the GlUS12 isolates had the same sequence.

All isolates had genomes of 5425 nucleotides (nt) and 144 nt-inverted terminal repeats, of which the 114 distal nt formed perfect hairpins. The location of open reading frames, TATA boxes, and splicing sites were all conserved compared to those of the original 1977 isolate (5). The highest protein sequence identity among the isolates was found for NS1 and its overlapping NS2 (both 99.3%), whereas NS3 had an identity of 94.4%, and the two ORFs, A and B, of the structural proteins had identities of 98.1 and 97.1%, respectively. Interestingly, phylogenetic analysis showed that the AdJP12 and GsCa12 clade diverged early from the European/North America clade, probably 20 years before the epidemics occurred simultaneously in 2009 in North America and Japan. This suggested another contributing factor to these outbreaks.

### Nucleotide sequence accession numbers.

The GenBank accession numbers are KF015274 for the AdDNV-AdEu04, KF015275 for the AdDNV-AdEu06, KF015276 for the AdDNV-AdEu07, KF015277 for the AdDNV-AdEu09, KF015278 for the AdDNV-AdNA09 and AdDNV-GlUS12, KF015279 for the AdDNV-AdJP12, and KF015280 for the AdDNV-GsCa12 isolates.
